# Successful endoscopic management of a severe esophagomediastinal fistula using a novel built-in catheter fixation device

**DOI:** 10.1055/a-2877-1837

**Published:** 2026-06-03

**Authors:** Xintong Jiang, Xueman Wang

**Affiliations:** 1 Endoscopy Center 74682 Shaoxing People’s Hospital (The First Affiliated Hospital, Shaoxing University)ShaoxingZhejiangChina

## Case description



**Video 1** Continuous drainage of purulent secretions from an esophagomediastinal fistula using a novel FTTC application. FTTC, fixed tube with two coils.


An esophagomediastinal fistula complicated by abscess formation is associated with high mortality and presents substantial therapeutic challenges. 1​ ​2​ Adequate drainage of the purulent material through a drainage tube positioned at the fistula opening is a critical component of effective management. 3​ However, a wide fistula opening predisposes the drainage tube to migration or dislodgement.

**Fig. 1:**
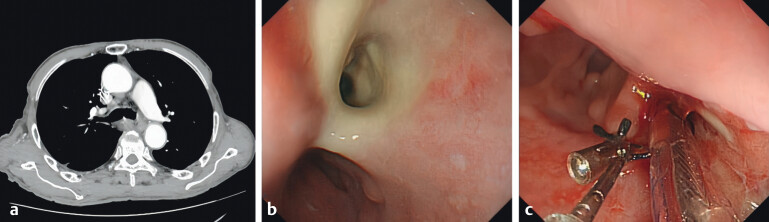
CT and endoscopic images: ( **a** ) Chest CT showing an esophagomediastinal fistula with mediastinal abscess formation. ( **b** ) Esophageal perforation with purulent material present. ( **c** ) Continuous drainage of purulent secretions from an esophagomediastinal fistula using a novel FTTC application. CT, computed tomography; FTTC, fixed tube with two coils.

**Fig. 2:**
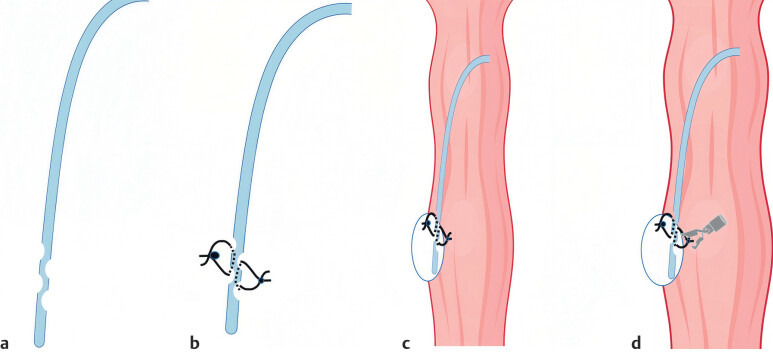
Schematic representation of the FTTC application: ( **a** ) use sterile surgical scissors to create symmetrical side holes in the drainage tube. ( **b** ) Two coils were symmetrically fixed on the drainage tube. ( **c** ) A drainage tube with a fixation device was inserted into the fistula opening. ( **d** ) A clip was used to secure the coil to the edge of the fistula opening, created using Fig Draw (www.figdraw.com), with publication license (ID: AWWPT35422). FTTC, fixed tube with two coils.

**Fig. 3:**
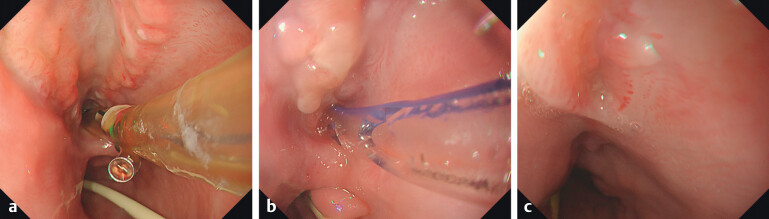
Endoscopic images obtained during the post-treatment follow-up: ( **a** ) A marked reduction in purulent discharge from the fistula opening and granulation tissue formation was observed at the site. ( **b** ) Significant narrowing of the fistula opening during the follow-up, with replacement of a thinner drainage tube. ( **c** ) Endoscopy confirmed that the fistula had healed.

A 74-year-old man was referred to our thoracic surgery department after computed tomography (CT) performed at another institution identified an esophagomediastinal fistula. The patient was diagnosed with nasopharyngeal carcinoma 5 months ago and was undergoing concurrent chemoradiotherapy and targeted therapy. The patient developed chest pain and dysphagia 1 week prior to presentation. A chest CT scan performed at another hospital revealed an esophagomediastinal fistula with associated mediastinal abscess formation ( Fig. 1a ). Endoscopic examination after admission revealed an esophageal perforation with purulent discharge ( Fig. 1b ). A tube was immediately positioned at the fistula site, and enteral nutritional support and anti-infective therapy were initiated. One week later, follow-up endoscopy showed the migration of the drainage tube into the esophageal lumen, accompanied by inadequate healing of the fistula orifice. Our team modified the drainage tube. Multiple side holes were created at the distal end of the drainage tube, and two coils were secured to these openings. The endoscopic clip was secured via the coil to the adjacent normal mucosa surrounding the fistula opening, thereby preventing tube displacement ( Fig. 1c and Video 1 ). We designated the modified drainage tube as a fixed tube with two coils (FTTC; Fig. 2 ). Postoperatively, we performed multiple daily irrigations through the drainage tube using normal saline (100 mL per session, every 2 h). A follow-up endoscopy performed 4 days later showed the FTTC to be in place, with a marked reduction in purulent discharge from the fistula opening and granulation tissue formation was observed at the site ( Fig. 3a ). Twelve days later, endoscopy revealed significant narrowing of the fistula opening, and the FTTC was subsequently replaced with a thinner drainage tube ( Fig. 3b ). The irrigation frequency was subsequently reduced to 100 mL per session, twice daily. Antibiotic therapy was maintained throughout this period to manage the infection, for a total duration of 3 weeks, and no additional parenteral nutrition was administered. After 30 days, endoscopy confirmed that the fistula had healed ( Fig. 3c ). The drainage tube and enteral feeding tube were removed, and the patient resumed oral intake with a cool liquid diet. The patient tolerated this well, without any significant discomfort.

We hypothesize that the displacement of the drainage tube may be associated with duct irrigation procedures, during which counteracting forces may cause the tube to dislodge from the fistula opening. By modifying the drainage tube design, we achieved continuous and unobstructed drainage from the fistula opening, thereby facilitating fistula healing. We propose that this FTTC has substantial potential for broader clinical applications.

## Declaration of interests

The authors declare that they have no known competing financial interests or personal relationships that could have appeared to influence the work reported in this paper. I would like to declare on behalf of my co-authors that the work described was original research that has not been published previously, and not under consideration for publication elsewhere, in whole or in part. All the authors listed have approved the manuscript that is enclosed.

Endoscopy_UCTN_Code_TTT_1AO_2AI

## References

[1] Bemelman W A Baron T H Endoscopic management of transmural defects, including leaks, perforations, and fistulae Gastroenterology 2018 154 07 1938 19460 10.1053/j.gastro.2018.01.067 29454791

[2] Righini C A Tea B Z Reyt E Cervical cellulitis and mediastinitis following esophageal perforation: A case report World J Gastroenterol 2008 14 09 1450 1452 10.3748/wjg.14.1450 18322964 PMC2693698

[3] Zhu R Zhang J Zheng Q A serious esophageal-mediastinal fistula successfully treated by endoscopic debridement and continuous irrigation Endoscopy 2025 57S01E323 E324 10.1055/a-2575-3622 40245943 PMC12020651

